# Osterix acetylation at K307 and K312 enhances its transcriptional activity and is required for osteoblast differentiation

**DOI:** 10.18632/oncotarget.9650

**Published:** 2016-05-26

**Authors:** Jianlei Lu, Shuang Qu, Bing Yao, Yuexin Xu, Yucui Jin, Kaikai Shi, Yifang Shui, Shiyang Pan, Li Chen, Changyan Ma

**Affiliations:** ^1^ Department of Developmental Genetics, Nanjing Medical University, Nanjing, P.R. China; ^2^ Department of Laboratory Medicine, the First Affiliated Hospital of Nanjing Medical University, Nanjing, P.R. China; ^3^ Molecular Endocrinology Laboratory, Department of Endocrinology, Odense University Hospital, Odense C, Denmark

**Keywords:** osterix, acetylation, CBP, osteoblast differentiation, Pathology Section

## Abstract

Osterix (Osx) is an essential transcription factor involved in osteoblast differentiation and bone formation. The precise molecular mechanisms of the regulation of Osx expression are not fully understood. In the present study, we found that in cells, both endogenous and exogenous Osx protein increased after treatment with histone deacetylase inhibitors Trichostatin A and hydroxamic acid. Meanwhile, the results of immunoprecipitation indicated that Osx was an acetylated protein and that the CREB binding protein (CBP), and less efficiently p300, acetylated Osx. The interaction and colocalization of CBP and Osx were demonstrated by Co-immunoprecipitation and immunofluorescence, respectively. In addition, K307 and K312 were identified as the acetylated sites of Osx. By contrast, HDAC4, a histone deacetylase (HDAC), was observed to interact and co-localize with Osx. HDAC4 was demonstrated to mediate the deacetylation of Osx. Moreover, we found that acetylation of Osx enhanced its stability, DNA binding ability and transcriptional activity. Finally, we demonstrated that acetylation of Osx was required for the osteogenic differentiation of C2C12 cells. Taken together, our results provide evidence that CBP-mediated acetylation and HDAC4-mediated deacetylation have critical roles in the modification of Osx, and thus are important in osteoblast differentiation.

## INTRODUCTION

Bone remodeling is a dynamic process that is maintained by the balance between osteoblast and osteoclast activity. Disruption of this balance will lead to certain pathological states, such as osteoporosis. Differentiation of osteoblasts from mesenchymal stem cells is controlled by a variety of transcription factors and signal molecules, such as ATF4, Runx2, Osterix (Osx or Sp7) and bone morphogenetic proteins (BMPs), fibroblast growth factors (FGFs), insulin-like growth factor 1 (IGF1) [1]. Most importantly, Osx is a critical regulator for the differentiation of pre-osteoblasts into functional osteoblasts [2].

Osx, which belongs to the Sp family, was identified as a C2H2-type zinc-finger-containing transcription factor in 2002 [3]. Osx-null mice died immediately after birth, with a complete absence of intramembranous and endochondral bone formation [3]. Mice with osteoblast-specific ablation of Osx were viable, but exhibited an osteopenia phenotype [4]. Mice with an Osx haploinsufficiency in their chondrocytes exhibited delayed growth of both trabecular and cortical bones [5]. In humans, genetic polymorphisms in the *Osx* gene locus are associated with low bone mineral density [6, 7]. Moreover, a frameshift mutation in *Osx* was reported to lead to osteogenesis imperfecta, revealing an important role of Osx in human bone development [8]. Therefore, determination of the regulatory mechanism of Osx expression could provide new insights into osteogenic differentiation and the treatment of bone related diseases.

Osx expression and activity are regulated by growth factors, transcription factors, protein interactions and epigenetics. For instance, BMP2 stimulates Osx expression in a Runx2-dependent or independent manner [9–11]. XBP1 (X-box binding protein 1), Runx2, Runx3 and Prx1 (Peroxiredoxin 1) positively or negatively regulate the transcription of *Osx* [12–15]. Additionally, NFATc1, p300, Brg1 and NO66 regulate the transcriptional activity of Osx via protein interactions [16–18]. Moreover, *Osx* expression can be regulated by certain miRNAs (microRNAs) via a post-transcriptional mechanism, such as miR-637, miR-214 and miR-143 [19–21]. Furthermore, the transcriptional activity and/or stability of Osx are regulated by post-translational modifications (PTMs), including phosphorylation and ubiquitination [17, 22–23]. Although these findings indicated significant progress, the exact regulatory mechanisms of Osx remain to be explored.

Lysine acetylation was first discovered on histones in 1968 [24]. Two counteracting enzymes, histone acetyltransferases (HATs) and histone deacetylases (HDACs), are responsible for acetylation catalysis [25]. HATs are responsible for adding an acetyl group from acetyl-CoA to an ε-amino group of certain lysine side chains of proteins. HDACs are responsible for removing acetyl groups and maintenance of the equilibrium of lysine acetylation. In addition to histones, many non-histone proteins, including transcription factors such as p53, GATA-1 and Runx2, have been identified as substrates of acetyltransferases [26–28]. HATs comprise three families: MYST, GNAT and p300/CBP [29]. CBP and p300 share ~90% homology in their HAT domains and ~93% homology in the bromodomain. Except for these two highly conserved domains, the homologies of other regions are much lower [30]. HDACs are classified into four groups [31]. In humans, HDAC4 belongs to class II, which is located in both the nucleus and cytoplasm. Lysine acetylation of non-histone proteins plays critical roles in the regulation of mRNA stability, DNA binding ability, transcriptional activity, cellular localization, protein stability, enzyme activity, protein–protein and protein–DNA interactions [24, 32]. As a key regulator of cellular events, protein acetylation has received more attention recently.

In the present study, we evaluated the effects of acetylation on the function of Osx. We demonstrated that Osx is acetylated and that CBP is the major HAT contributing to Osx acetylation. In addition, we identified K307 and K312 as the acetylated sites of Osx and demonstrated that CBP interacted and co-localized with Osx. In addition, we demonstrated that HDAC4 is involved in the deacetylation of Osx. Functionally, we found that acetylation of Osx enhances its stability, DNA binding ability and transcriptional activity. Finally, we demonstrated that acetylation of Osx is required for the osteogenic differentiation of C2C12 cells. Taken together, our results provide the first evidence that CBP-mediated acetylation and HDAC4 mediated deacetylation have critical roles in the modification of Osx, and are thus important in osteoblast differentiation.

## RESULTS

### The Osx protein is acetylated

**Figure 1:**
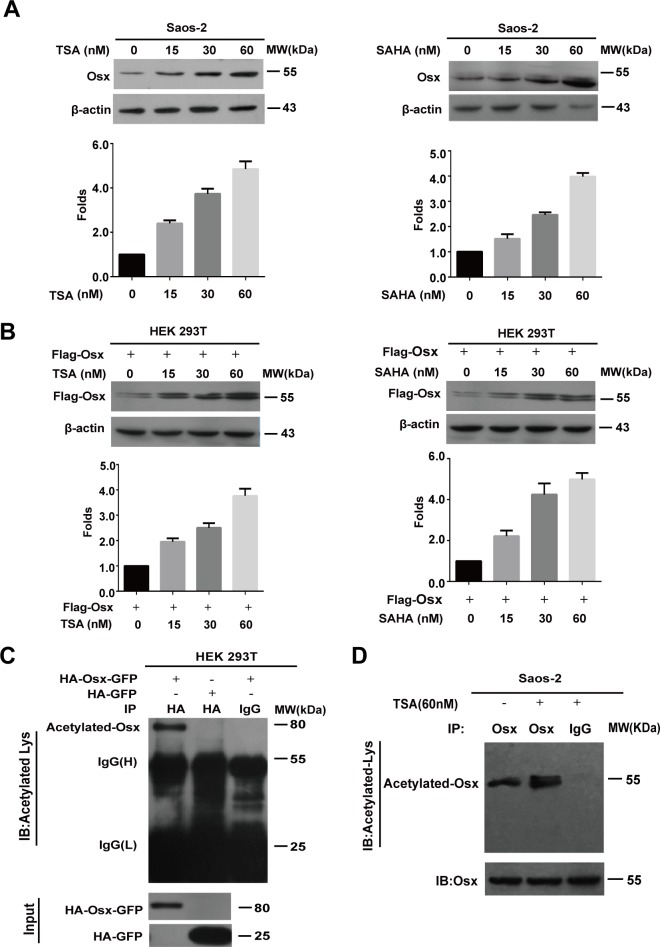
The Osx protein is acetylated **A.** Saos-2 cells were treated with TSA (0, 15, 30, 60 nM) or SAHA (0, 15, 30, 60 nM) for 24 h. Endogenous Osx protein was detected by western blotting with an anti-Osx antibody. β-Actin served as a loading control. The protein levels of Osx in the upper panel were determined by densitometry (lower). **B.** HEK 293T cells were transfected with Flag-Osx plasmids and then treated with TSA (0, 15, 30, 60 nM) or SAHA (0, 15, 30, 60 nM) for 24 h. Exogenous Osx protein was detected by western blotting with an anti-Flag antibody. β-Actin served as a loading control. The protein levels of Osx in upper panel were determined by densitometry (lower panel). **C.** HEK 293T cells were transiently transfected with HA-Osx-GFP or HA-GFP expression plasmids. The cell lysates were immunoprecipitated with an anti-HA antibody and then blotted with an anti-Acetylated-Lys antibody. HA-Osx-GFP and HA-GFP protein were detected by western blotting analysis with an anti-HA antibody. **D.** Saos-2 cells were treated with or without TSA (60 nM) for 24 h. The cell lysates were immunoprecipitated with an anti-Osx antibody (rabbit polyclonal antibody) and then blotted with an anti-Acetylated-Lysine antibody (mouse monoclonal antibody). Osx protein was detected by western blotting analysis with an anti-Osx antibody. Results are shown for one of three independent experiments.

To determine whether acetylation modification is involved in the regulation of Osx, we firstly examined the effects of HDAC inhibitors (HDACI) on the expression of Osx. Saos-2 cells, which express endogenous Osx, or HEK 293T cells, which overexpressed Flag-tagged Osx, were treated by Trichostatin A (TSA) or hydroxamic acid (SAHA). Western blotting showed that the endogenous Osx protein increased in a dose dependent manner after TSA or SAHA treatment (Figure 1A). Meanwhile, the exogenous Osx protein also increased in a dose dependent manner after TSA or SAHA treatment (Figure 1B). Moreover, we found that TSA and SAHA treatment had no obvious effects on the mRNA levels of *Osx* (Supplemental Figure S1A-D). These results suggest that acetylation modification might be involved in the post-translational regulation of Osx expression.

To further verify that Osx is acetylated in cells, immunoprecipitation (IP) assays with an anti-acetyl-lysine specific antibody were performed. To overcome the problem of the similar molecular weight of Osx and IgG (H), we constructed a plasmid that expressed Osx fused to GFP (HA-Osx-GFP), and overexpressed it in HEK 293T cells. As shown in Figure 1C, the acetylated form of Osx was detected using the anti-acetyl-lysine specific antibody in HEK 293T cells that overexpresssed HA-Osx-GFP. To determine whether the endogenous Osx protein is acetylated, we treated Saos-2 cells with or without TSA (60 nM) for 24 h and the cell lysates were then used for IP assay. As shown in Figure 1D, the acetylated Osx was detected in Saos-2 cells and TSA treatment increased the acetylation level of the endogenous Osx. Together, these data indicate that Osx is a protein regulated by acetylation.

### Osx acetylation is mediated by p300/CBP

**Figure 2:**
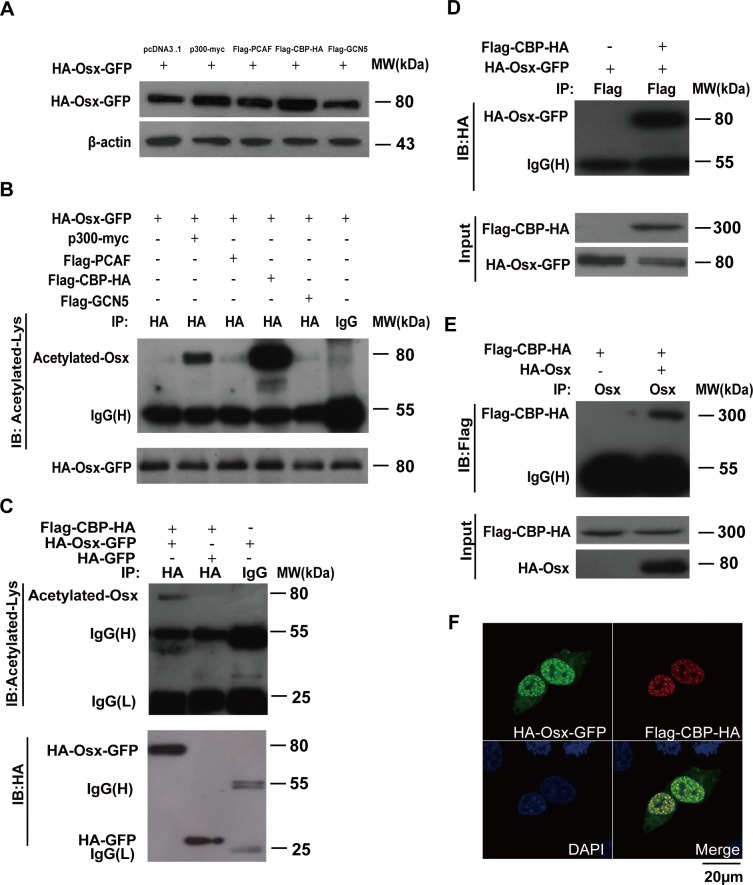
Osx acetylation is mediated by CBP/p300 **A.** HEK 293T cells were transiently co-transfected with HA-Osx-GFP and p300-myc, Flag-PCAF, Flag-CBP-HA, or Flag-GCN5 expression vectors. pcDNA3.1 empty vector co-transfection was used as a control. The Osx protein was detected by western blotting with an anti-HA antibody. β-Actin served as a loading control. **B.** The cell lysates of the above-transfected cells were immunoprecipitated with an anti-HA antibody and then blotted with an anti-Acetylated-Lys antibody. HA-Osx-GFP protein was detected by western blotting with an anti-HA antibody. **C.** HEK 293T cells were transiently co-transfected with Flag-CBP-HA and HA-Osx-GFP or HA-GFP expression plasmids, the cell lysates were immunoprecipitated with an anti-HA antibody and then blotted with an anti-Acetylated-Lys antibody. HA-Osx-GFP and HA-GFP protein were detected by western blotting with an anti-HA antibody. **D.** HEK 293T cells were transiently transfected with HA-Osx-GFP alone or together with the Flag-CBP-HA expression plasmid, the cell lysates were immunoprecipitated with an anti-Flag antibody and then blotted with an anti-HA antibody. Flag-CBP-HA and HA-Osx-GFP protein were detected by western blotting with an anti-HA antibody. **E.** HEK 293T cells were transiently transfected with Flag-CBP-HA alone or together with HA-Osx expression plasmid, the cell lysates were immunoprecipitated with an anti-Osx antibody and then blotted with an anti-Flag antibody. Flag-CBP-HA and HA-Osx protein were detected by western blotting with an anti-HA antibody. **F.** HEK 293T cells were transiently co-transfected with HA-Osx-GFP and Flag-CBP-HA expression plasmids. The localization of Osx was visualized as green fluorescence, and CBP was visualized by immunostaining with an anti-Flag antibody (red). Nuclei were visualized using 4, 6-diamidino-2-phenylindole (DAPI) staining (blue). Experiments were repeated at least three times.

The above data prompted us to identify the histone acetyltransferases that are responsible for Osx acetylation. We transfected p300, PCAF, CBP and GCN5 expression plasmids, separately, together with the HA-Osx-GFP construct into HEK 293T cells. The cell lysates were analyzed by immunoblotting to examine the Osx protein level. As shown in Figure 2A, both p300 and CBP increased the Osx protein level, although by different amounts, hinting that these two enzymes might be responsible for Osx acetylation. The expressions of p300, PCAF, CBP and GCN5 are shown in Supplemental Figure S2A. In addition, we found that there was no obvious effect of histone acetyltransferases on the mRNA expression of *Osx* (Supplemental Figure S2B). The same cell lysates were analyzed with an anti-HA antibody for IP and an anti-acetyl-lysine specific antibody for immunodetection. The results showed that both CBP and p300 increased the level of Osx acetylation (Figure 2B). However, CBP's promotion of Osx acetylation was much stronger than that of p300 (Figure 2B). To rule out the interference of GFP, HA-Osx-GFP or HA-GFP was co-transfected with a CBP expression plasmid into HEK 293T cells followed by an IP assay. As shown in Figure 2C, acetylated Osx was detected in the lysates of cells co-transfected with HA-Osx-GFP and CBP, while no acetylated GFP was detected in the lysates of cells co-transfected with HA-GFP and CBP. Taken together, these results suggested that CBP is the major acetyltransferase that mediates Osx acetylation.

To test whether CBP interacts with Osx *in vivo*, cell extracts from HEK 293T cells ectopically expressing Flag-CBP-HA and HA-Osx-GFP were used in a Flag IP assay. After probing the immunocomplexes using an anti-HA antibody, the Osx protein was detected in CBP immunoprecipitates (Figure 2D). Conversely, CBP protein was detected in Osx immunoprecipitates (Figure 2E). These results revealed an interaction between CBP and Osx. Corroborating these data, CBP was observed to be co-localized with Osx in the nucleus using immunofluorescence staining (Figure 2F).

### CBP acetylates Osx at K307 and K312

**Figure 3:**
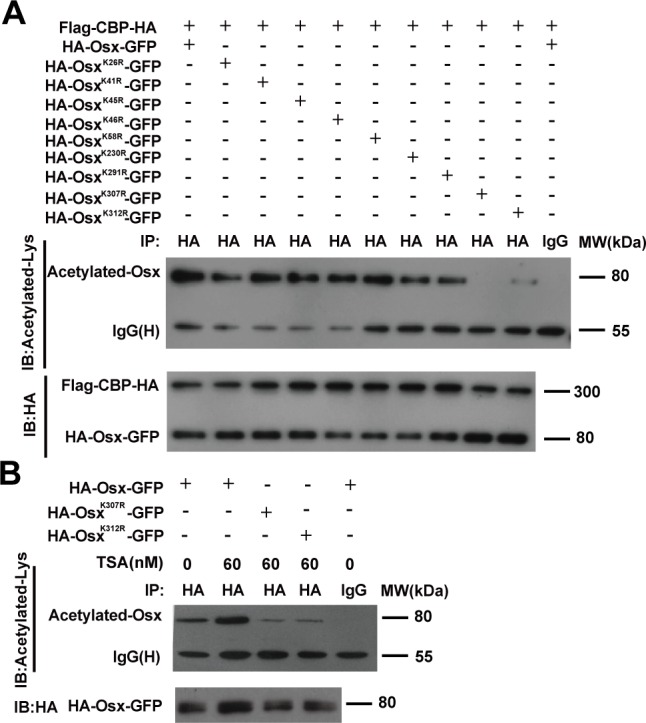
Identification of the acetylated site(s) of Osx mediated by CBP **A.** HEK 293T cells were transiently co-transfected with Flag-CBP-HA and HA-Osx-GFP expression plasmids or K26R, K41R, K45R, K46R, K58R, K230R, K291R, K307R, K312R mutants. The cell lysates were immunoprecipitated with an anti-HA antibody and then blotted with an anti-Acetylated-Lys antibody. Flag-CBP-HA and HA-Osx-GFP protein were detected by western blotting with an anti-HA antibody. **B.** HEK 293T cells were transiently transfected with HA-Osx-GFP expression plasmid or the K307R, K312R mutants. 24 h after transfection, the cells were treated with or without 60 μM TSA for 24 h. The cell lysates were immunoprecipitated with an anti-HA antibody and then blotted with an anti-Acetylated-Lys antibody. HA-Osx-GFP protein were detected by western blotting with an anti-HA antibody. Experiments were repeated at least three times.

In a previous study, we generated series Osx mutants with lysine-to-arginine (K to R) substitutions to identify the ubiquitination sites of Osx [22]. Using luciferase reporter assays, we found that the K26R, K41R, K45R, K46R, K58R and K230R mutants increased the transactivation activities of Osx; however, K291R, K307R and K312R mutants decreased the transactivation activities of Osx [22]. Besides providing specific sites for ubiquitination, lysine also provides sites for acetylation. To test whether the acetylation sites of Osx resided in these nine lysine residues, these Osx mutants were co-transfected with the CBP expression plasmid into HEK 293T cells. The cell lysates were immunoprecipitated with an anti-HA antibody and western blotted with an anti-acetyl-lysine antibody. As shown in Figure 3A, the acetylation of Osx mediated by CBP was decreased markedly in cells transfected with the Osx^K307R^ and Osx^K312R^ mutants, indicating that K307 and K312 are the acetylated sites of Osx. Moreover, TSA treatment increased the acetylation level of Osx and had little effect on the acetylation level of the Osx^K307R^ and Osx^K312R^ mutants (Figure 3B).

### HDAC4 mediates Osx deacetylation

**Figure 4:**
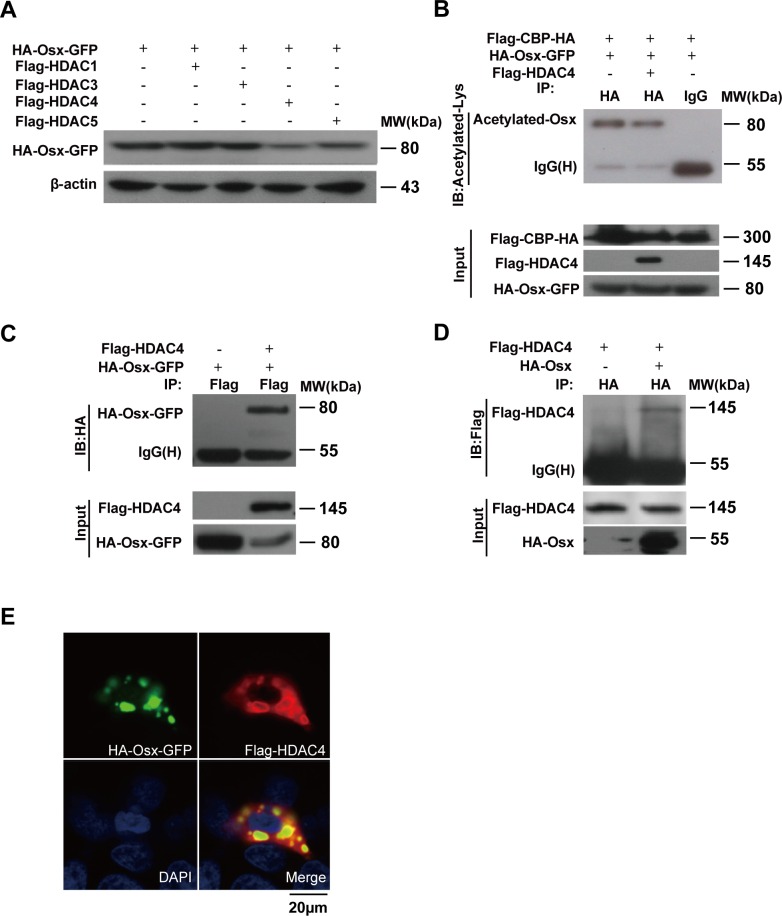
HDAC4 mediates Osx deacetylation **A.** HEK 293T cells were transiently transfected with the HA-Osx-GFP expression plasmid alone or together with Flag-HDAC1, Flag-HDAC3, Flag-HDAC4, or Flag-HDAC5 expression vectors. The Osx protein was detected by western blotting with an anti-HA antibody. β-Actin served as a loading control. **B.** HEK 293T cells were transiently co-transfected with Flag-CBP-HA, HA-Osx-GFP and Flag-HDAC4 or not, the cell lysates were immunoprecipitated with an anti-HA antibody and then blotted with an anti-Acetylated-Lys antibody. Flag-CBP-HA and Flag-HDAC4 protein were detected by western blotting with an anti-Flag antibody. HA-Osx-GFP protein was detected with an anti-HA antibody. **C.** HEK 293T cells were transiently transfected with HA-Osx-GFP alone or together with Flag-HDAC4 expression plasmid, the cell lysates were immunoprecipitated with an anti-Flag antibody and then blotted with an anti-HA antibody. Flag-HDAC4 and HA-Osx-GFP protein were detected by western blotting with an anti-Flag and anti-HA antibody, respectively. **D.** HEK 293T cells were transiently transfected with Flag-HDAC4 alone or together with the HA-Osx expression plasmid, the cell lysates were immunoprecipitated with an anti-HA antibody and then blotted with an anti-Flag antibody. Flag-HDAC4 and HA-Osx protein were detected by western blotting with an anti-Flag and anti-HA antibody, respectively. **E.** HEK 293T cells were transiently co-transfected with HA-Osx-GFP and Flag-HDAC4 expression plasmids. The localization of Osx was visualized as green fluorescence, the localization of HDAC4 was visualized by immunostaining with an anti-Flag antibody (red). Nuclei were visualized by DAPI staining (blue). Experiments were repeated at least three times.

Lysine acetylation is a reversible protein modification, which is controlled by two opposing types of enzymes families: HATs and HDACs. We next set out to investigate whether any of the HDACs (HDAC1, −3, −4, and −5) is capable of deacetylating Osx. As shown in Figure 4A, HDAC4 overexpression led to a decrease in the Osx protein level, indicating that HDAC4 might be involved in the deacetylation of Osx. The expressions of HDAC1, −3, −4, and −5 are shown in Supplemental Figure S3A. In addition, we found that there was no obvious effect of the HDACs on mRNA expression of Osx (Supplemental Figure S3B). To further confirm the effect of HDAC4 on Osx acetylation, we performed an IP assay using cell lysates from HEK 293T transfected with CBP and Osx, with or without HDAC4 expression plasmids. The results indicated that HDAC4 decreased the level of Osx acetylation mediated by CBP (Figure 4B), providing evidence that HDAC4 mediated Osx deacetylation. Furthermore, HEK 293T cells were co-transfected with Flag-HDAC4 and HA-Osx-GFP or HA-Osx expression plasmids, and the cell lysates were then analyzed using Co-IP assays. Osx was detected in HDAC4 immunoprecipitates (Figure 4C). Conversely, HDAC4 was detected in Osx immunoprecipitates (Figure 4D). In addition, an immunofluorescence staining assay showed that HDAC4 co-transfection led to the export of Osx from nucleus to the cytoplasm and that HDAC4 was co-localized with Osx in the cytoplasm (Figure 4E). These results revealed that HDAC4 physically interacts with Osx.

### Acetylation increases the stability of Osx

**Figure 5:**
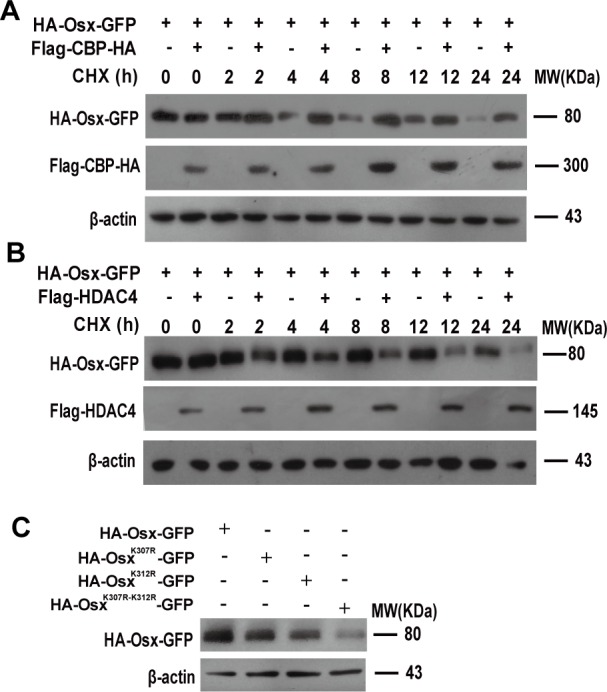
Acetylation increases the stability of Osx **A.** HEK 293T cells were transfected with HA-Osx expression plasmids alone or together with Flag-CBP-HA and then treated with 80 μM CHX for the indicated periods. Osx and CBP proteins were detected by western blotting with an anti-HA antibody. **B.** HEK 293T cells were transfected with HA-Osx expression plasmids alone or together with Flag-HDAC4 and then treated with 80 μM CHX for the indicated periods. Osx and HDAC4 proteins were detected by western blotting with an anti-HA and anti-Flag antibody, respectively. **C.** HEK 293T cells were transfected with HA-Osx-GFP, HA-Osx^K307^-GFP, HA-Osx^K312^-GFP and HA-Osx^K307-K312^-GFP expression plasmids, respectively. 48 h after transfection, the Osx protein was detected with an anti-HA antibody. β-Actin served as a loading control. Results are shown for one of three independent experiments.

Our previous studies revealed that Osx is an unstable protein and the ubiquitin proteasome pathway is involved in the regulation of Osx stability [22]. To assess the effect of acetylation on Osx stability, we transfected HEK 293T cells with Osx constructs alone or together with CBP or HDAC4 expression plasmids and then treated the cells with the protein synthesis inhibitor cycloheximide (CHX). Compared with Osx transfection alone, CBP co-transfection markedly delayed the degradation of Osx (Figure 5A). Conversely, HDAC4 co-transfection markedly accelerated the degradation of Osx (Figure 5B). In addition, the stabilities of Osx^K307R^, Osx^K312R^ and Osx^K307R-K312R^ were decreased compared with Osx (WT) (Figure 5C). The similar transfection efficiency of the Osx^K307R^, Osx^K312R^ and Osx^K307R-K312R^ expression plasmids was confirmed in Supplemental Figure S4.

### Acetylation increases the DNA binding activity of Osx

**Figure 6:**
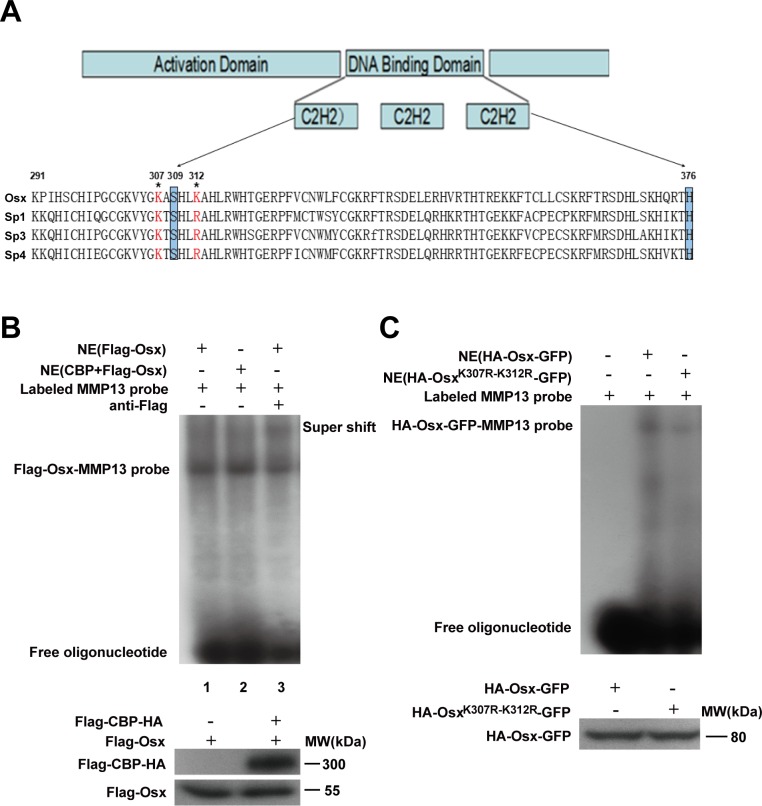
Acetylation increases the DNA binding activity of Osx **A.** Evolutionary conservation analysis of the acetylation sites in Osx. Schematic representation of Osx, its functional domains, the two acetylated lysine residues that were mutated to arginines and a partial polypeptide sequence alignment of Osx with Sp1, Sp3 and Sp4. The highlighted boxes indicate the zinc-finger domain (from amino acid 309 to 376). The acetylated lysines are indicated by asterisks. **B.** Nuclear extracts (NE) from HEK 293T cells transfected with Flag-Osx expression plasmid alone or together with Flag-CBP-HA were incubated with a ^32^P-labeled Osx-binding oligonucleotide (MMP-13 probe) in the absence (lane 1 and 2) or presence of anti-Flag antibodies (lane 3). The reaction mixtures were analyzed by EMSA. Osx and CBP proteins were detected by western blotting with an anti-Flag antibody. **C.** Nuclear extracts from HEK 293T cells transfected with HA-Osx-GFP or HA-Osx^K307R-K312R^-GFP expression plasmids were incubated in reaction mixtures containing ^32^P-labeled MMP-13 probe, resolved by electrophoreses and visualized by autoradiography. Osx and Osx^K307R-K312R^ proteins were detected by western blotting with an anti-HA antibody. Experiments were repeated at least three times.

We then investigated the effect of acetylation on the DNA binding activity of Osx. First, we determined whether K307 and K312 were conserved between different members of the Sp family. As shown in Figure 6A, K307 is conserved across several members, while K312 is not conserved. K307 is just adjacent to the C2H2 DNA binding domain (from amino acid 309 to 376) and K312 is located at the N-terminus of the DNA binding domain of Osx (Figure 6A). Electrophoretic mobility shift assays (EMSAs) showed that CBP and Flag-Osx co-expression (Figure 6B, lane 2) markedly increased the DNA binding ability of Osx compared with Flag-Osx expression plasmid transfection alone (Figure 6B, lane 1). A supershift effect was observed by the addition of a Flag antibody, indicating the specificity of Osx binding (Figure 6B, lane 3). Moreover, the DNA binding activity of Osx^K307R-K312R^ was decreased compared with the wild-type Osx (Figure 6C).

### Acetylation increases the transactivation activity of Osx and deacetylation of Osx impairs osteoblast differentiation

**Figure 7:**
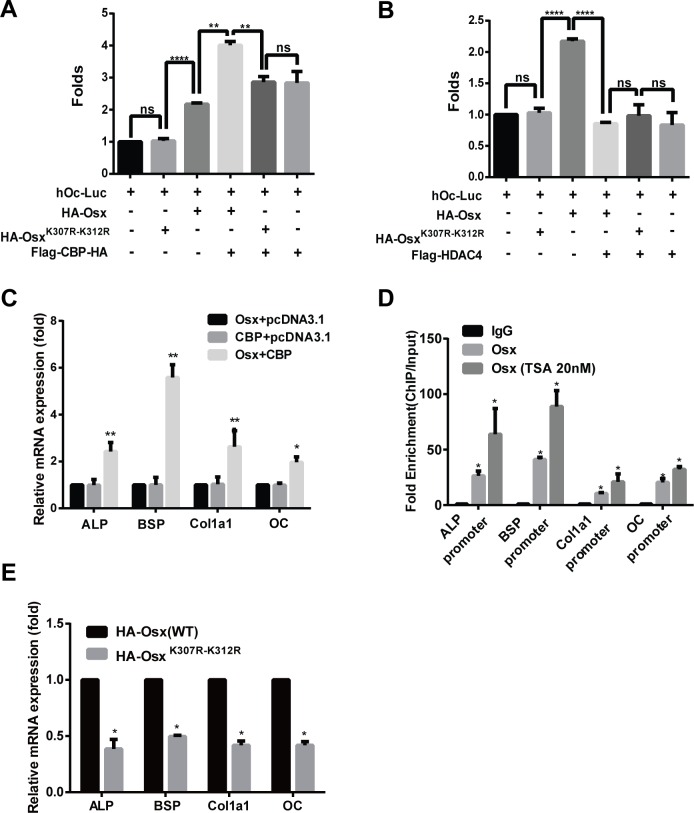

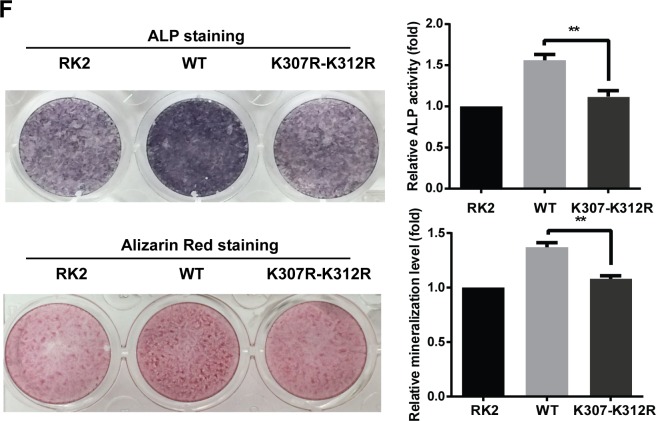
Acetylation increases the transactivation activity of Osx and deacetylation of Osx impairs osteoblast differentiation **A.** HEK 293T cells were transfected with a hOc-Luc reporter, β-gal expression vector and together with or without Flag-Osx, HA-Osx^K307R-K312R^ and Flag-CBP-HA expression plasmids. **B.** HEK 293T cells were transfected with the hOc-Luc reporter, β-gal expression vector, and together with or without Flag-Osx, HA-Osx^K307R-K312R^ and Flag-HDAC4 expression plasmids. Relative luciferase activities of A and B were measured 36 h after transfection and normalized to the β-gal activity. **C.** MC3T3 E1 cells were transfected with Flag-Osx and Flag-CBP-HA expression plasmids alone or together. 36 h after transfection, *ALP*, *BSP*, *Col1a1* and *OC* mRNAs were measured using real-time PCR. β-Actin was used as the internal control. **D.** MC3T3 cells were treated with rhBMP-2 (100 ng/ml) for 24 h followed by TSA (20 nM) for 24 h or not. ChIP assay were performed to examine the levels of Osx on the promoter of *ALP*, *BSP*, *Col1a1*, and *OC*. Data are presented as a percentage of input after subtracting control IgG values. **E.** C2C12 cells were transfected with HA-Osx(WT) or HA-Osx^K307R-K312R^ expression plasmids. 36 h after transfection, *ALP*, *BSP*, *Col1a1*, and OC mRNAs were measured using real-time PCR. β-Actin was used as the internal control. **F.** C2C12 cells were transiently transfected with empty RK2 vector, the HA-Osx(WT) plasmid or HA-Osx^K307R-K312R^ mutants. 24 h after transfection, the cells were incubated with BMP2 (100 ng/ml). ALP activity was examined by ALP staining 4–5 days later, or mineralization was assessed using Alizarin Red staining 10-12 days later. Representative images of three independent experiments are shown (left panel). Quantification of ALP activity and mineralization is shown in the right panel. Results are the mean ± S.D. of three independent experiments; *P <0.05, ** p < 0.01, *** p < 0.001 and **** p < 0.0001.

Finally, we examined the effects of acetylation on the transactivation activity of Osx and on osteoblast differentiation. Using the reporter assay, the luciferase activity driven by the Osx binding site was remarkably increased by CBP co-expression, and this increase was attenuated when the acetylated sites K307 and K312 were mutated (Figure 7A). Meanwhile, we found that CBP alone activated the OC promoter activity (Figure 7A). The luciferase activity was markedly decreased by HADC4 co-expression, and HDAC4 had no obvious effect on the luciferase activity when the acetylated sites K307 and K312 were mutated (Figure 7B). Together, these data indicate that acetylation potentiates the transcriptional activity of Osx. To confirm this further, we transfected expression plasmids of Osx and CBP, alone or together, into preosteoblast cells MC3T3 E1, and the mRNA levels of Osx target genes including *ALP* (alkaline phosphatase), *BSP* (bone sialoprotein), *Colla1* (collagen I) and *OC* (osteocalcin) were measured by real-time PCR assays. As shown in Figure 7C, CBP co-transfection significantly increased the mRNA expressions of *ALP*, *BSP*, *Colla1* and *OC* compared with Osx transfection alone. To explore the mechanism by which acetylation of Osx enhances its transcription activity, we performed Chromatin immunoprecipitation (ChIP) assay. As shown in Figure 7D, the occupancy of Osx at the chromatin fragments of the promoter of *ALP*, *BSP*, *Col1a1*, and *OC*, was significantly increased in MC3T3 E1 cells treated with TSA compared to the untreated cells. These data suggest that acetylation of Osx enhances its transcription activity by increasing its binding to the promoter of the target genes.

To evaluate the role of Osx acetylation in osteoblastic differentiation further, we transiently transfected HA-Osx(WT) or the HA-Osx^K307R-K312R^ mutant construct into C2C12 cells, a typical pluripotent mesenchymal precursor cell line that has the potential to differentiate into osteoblasts, chondroblasts, myoblasts, or adipocytes. As shown in Figure 7E, the expressions of the marker genes of osteoblast differentiation were obviously decreased in C2C12 cells transfected with Osx^K307R-K312R^ compared with the cells transfected with Osx(WT). Simultaneously, ALP staining and Alizarin Red staining (ARS) revealed that the ALP activity and mineralization in C2C12 cells transfected with Osx^K307R-K312R^ mutants were markedly reduced compared with the cells transfected with the Osx(WT) plasmids (Figure 7F). The same results were observed in MC3T3 E1 cells (Supplemental Figure S5). Taken together, these data proved that acetylation increased the Osx transcriptional activity and is required for Osx to promote osteoblast differentiation.

## DISCUSSION

In this study, we presented evidence that acetylation plays an important role in the function of Osx. We found that the transcriptional coactivator CBP interacts with Osx, resulting in acetylation of Osx on two lysine residues in its C terminus. Furthermore, HDAC4 interacts with and deacetylates Osx. Acetylation of these two lysine residues stabilizes Osx and enhances its DNA binding activity and transcriptional activity. Moreover, we demonstrated that acetylation of lysine 307 and 312 is required for Osx to promote osteoblast differentiation.

Although CBP and p300 acetylate and interact with overlapping sets of proteins, accumulating evidence suggests that CBP and p300 possess discrete functions in cells. In mice, heterozygous inactivation of CBP and p300 led to diverse phenotypes, and neither of them can compensate for the loss of the other [33–35]. In humans, mutations in the *CBP* gene have been associated with Rubinstein-Taybi syndrome and fetal alcohol syndrome, while deficiencies in p300 have been associated with multiple cancers [33, 36–37]. We found that Osx was more efficiently acetylated by CBP than by p300, confirming the hypothesis that CBP and p300 have different functions.

Studies have revealed that HDAC4 is a central regulator of chondrocyte hypertrophy and skeletogenesis [38, 39]. *HDAC4* gene knockout mice showed premature ossification. By contrast, overexpression of HDAC4 *in vivo* resulted in inhibition of chondrocyte hypertrophy, with delayed ossification. HDAC4 regulates chondrocyte hypertrophy and endochondral bone formation by interacting with Runx2 and inhibiting its activity [38, 39]. Our results revealed that HDAC4 interacts with Osx and mediates its deacetylation. These data reveal another mechanism by which HDAC4 exerts its important roles in bone development.

Like phosphorylation, acetylation is a universal modification of proteins [40]. More than 2000 non-histone proteins, including transcription factors, have been identified as acetylated [41]. Acetylation can either enhance or decrease the DNA binding and transcriptional activity of a transcription factor. For instance, acetylation of p53 by p300/CBP promotes its DNA binding and transcriptional activity [42–44]. Whereas, acetylation of the Yin Yang 1 (YY1) protein by p300/CBP resulted in decreased DNA binding [45]. Stimulation or inhibition of the activity of a given transcription factor by acetylation depends on the site of modification. For example, the activities of transcription factor HMG-A1 are stimulated by lysine 71 acetylation, while they are inhibited by lysine 65 acetylation [46, 47]. Generally, if the acetylation sites lie in a DNA-binding domain, acetylation will repress DNA-binding and if they are adjacent to a DNA-binding domain, it may activate DNA binding [48, 49]. Here, we identified K307 and K312 as the acetylated sites of Osx. K307 is just adjacent to the C2H2 DNA binding domain (from amino acid 309 to 376) and K312 is located at the N-terminus of the DNA binding domain of Osx. We speculated that acetylation increases the DNA binding activity of Osx, possibly by altering its conformation. Further study is needed to investigate the structural changes of Osx after acetylation.

Acetylation can affect protein–protein, protein–DNA and protein–RNA interactions, as well as subcellular distribution and protein stability, by neutralizing the positive charge of the lysine side chain or impairing the formation of hydrogen bonds [50]. Increasing a protein's stability by acetylation might act through direct competition for ubiquitination of the same lysine residues or via other mechanisms that inhibit ubiquitin-dependent degradation [51, 52]. In our previous studies, we identified K58 and K230 as the ubiquitination sites of Osx, not K307 and K312 [22]. We speculated that acetylation increases Osx protein stability via an indirect mechanism, such as influencing the interaction between the ubiquitination-related enzymes and Osx by changing Osx's conformation, thereby inhibiting ubiquitin-dependent degradation of Osx.

In summary, we demonstrated that Osx is acetylated and that this modification plays important roles in regulating the protein stability, DNA binding activity and transcriptional activity of Osx, as well as in osteoblast differentiation. Further studies are needed to understand which signals control the acetylation and deacetylation of Osx, as well as any crosstalk between acetylation and other post-translational modifications, such as phosphorylation and ubiquitination of Osx.

## MATERIALS AND METHODS

### Cell culture

Saos-2 cells were maintained in McCoy's 5A medium supplemented with 10% fetal bovine serum (FBS). MC3T3-E1 cells were maintained in alpha minimum essential medium (α-MEM) supplemented with 10% FBS. HEK 293T cells were cultured in high glucose Dulbecco's modified Eagle's medium (DMEM) supplemented with 10% FBS. C2C12 cells were maintained in DMEM supplemented with 15% FBS. All cells were purchased from American Type Culture Collection (ATCC, Manassas, VA, USA) and cultured in the presence of 100 units/ml penicillin and 100 μg/ml streptomycin at 37°C in 5% CO_2_.

### Plasmids, chemicals and antibodies

pCMVβ-p300-myc, pCI-Flag-PCAF, pcDNA3β-Flag-CBP-HA, pAdEasy-Flag-GCN5, pcDNA3.1-Flag-HDAC1, pcDNA3.1-Flag-HDAC3, pcDNA3.1-Flag-HDAC4, and pcDNA3.1-Flag-HDAC5 expression plasmids were obtained from Addgene (Cambridge, MA, USA). RK2-HA-Osx-GFP, RK2-HA-Osx(WT) and Lys (K) to Arg (R) mutants of Osx were generated as descripted previously [35]. The RK2-HA-Osx^K307R-K312R^ mutant was generated using Fast Mutagenesis System (TransGen Biotech, Beijing, P. R. China) using RK2-HA-Osx(WT) as a template. CHX, TSA and SAHA were purchased from Sigma Aldrich (St. Louis, MO, USA). Protease inhibitors were obtained from Roche Diagnostics (Mannheim, Germany). Recombinant BMP2 was purchased from R&D Systems (Minneapolis, MN, USA). The anti-Osx antibody was obtained from Abcam (Cambridge, MA, USA). The anti-Flag antibody was obtained from Sigma. The anti-HA antibody was obtained from Roche (Roche Diagnostics, Indianapolis, IN, USA). The anti-acetylated-lysine antibodies (rabbit polyclonal antibody and mouse monoclonal antibody) were purchased from Cell Signaling Technology (Danvers, MA, USA). EasyBlot anti-Rabbit IgG Kit (Optimized for Prot A/G) was purchased from Genetex (Irvine, CA, USA). Antibody against β-actin, anti-rabbit, anti-rat and anti-mouse IgG antibody were obtained from Bioworld Technology (Minneapolis, MN, USA). Lipofectamine 2000, Goat anti-rabbit IgG and Alexa Fluor 594 antibodies were purchased from Invitrogen (Carlsbad, CA, USA). EZ-ChIP kit and Protein-G agarose beads were from Millipore (Darmstadt, Germany).

### Western blotting

Western blotting was performed as previously described [22, 23]. Briefly, cells were lysed in RIPA buffer plus protease inhibitors, separated via SDS-PAGE, transferred to PVDF membrane, immunoblotted, and detected with the ECL reagent.

### Co-immunoprecipitation (Co-IP) assay

HEK 293T cells were co-transfected with HA-Osx-GFP and pcDNA3β-Flag-CBP-HA or pcDNA3-Flag-HDAC4 expression plasmids. After 48 h of transfection, cells were washed twice with ice-cold phosphate-buffered saline (PBS) and then lysed with RIPA buffer (50 mM Tris-HCl, pH 7.5, 150 mM NaCl, 1% NP-40, 0.5% sodium deoxycholate and 0.1% SDS). The cell lysates were incubated with anti-HA, anti-Osx or anti-Flag antibody at 4°C overnight. The mixture was then incubated with protein-G agarose beads at 4°C for 4 h. The beads were collected and washed three times by RIPA buffer. The beads were resuspended in SDS gel-loading buffer and analyzed by immunoblotting with anti-HA or anti-Flag antibodies.

For Co-IP of endogenous proteins, Saos-2 cells were treated with or without TSA (60 nM) for 24 h and then were lysed. The Co-IP was performed as above using anti-Osx antibody (rabbit polyclonal antibody) to precipitate the immunocomplexes and the anti-Acetylated-Lysine antibody (mouse monoclonal antibody) to perform western blot analysis.

### Immunofluorescence and confocal microscopy

HEK 293T cells were plated onto a six-well plate with glass bottom inserts. The cells were co-transfected with HA-Osx-GFP and Flag-CBP-HA or Flag-HDAC4 expression plasmids. 30 h after transfection, the cells were fixed with 4% paraformaldehyde for 20 min at room temperature. After washing twice with PBS, the cells were permeabilized with PBS containing 0.5% Triton X-100 for 10 min at 4°C, then treated with blocking buffer containing 4% bovine serum albumin (BSA) for 45 min at room temperature. Immunostaining was conducted with anti-Flag antibody overnight at 4°C, followed by Alexa Fluor 594-conjugated secondary antibodies for 1 h at room temperature. Finally, the cells were mounted in 90% glycerol containing 4, 6-diamidino-2-phenylindole (DAPI), and examined using a confocal laser-scanning microscope (Zeiss LSM710). The images were analyzed using ZEN2011 software.

### EMSA

Nuclear extracts of transfected HEK 293T cells were prepared using NE-PER Nuclear and Cytoplasmic Extraction Reagents and Halt^TM^ Protease Inhibitor Cocktail Kit (Pierce Biotechnology, Rockford, IL, USA), as recommended by the manufacturer. EMSA were performed using the Gel Shift Assay System (Promega Corp., Madison, WI, USA) according to the instructions of the manufacturer. Oligonucleotide probes were annealed and labeled with [r-^32^P] ATP and T4-polynucleotide kinase (NEB, Ipswich, MA, USA). Nuclear extracts of cells were incubated with the ^32^P-labeled probe in a total volume of 10 μl, as recommended by the manufacturer. The protein–DNA complexes were separated by 5% PAGE. Signals were recorded on X-ray film. The sequence of the oligonucleotide containing an Osx-binding sequence from the MMP-13 promoter was as follows: 5′-AGGAAGTTAACACACACCCC AAAGTGGTGACTCATC-3′. For the supershift experiments, anti-Flag antibody was added during pre-incubation.

### Luciferase reporter assay

HEK 293T cells were co-transfected with the hOc-Luc reporter, β-gal expression vector, the Flag-Osx and Flag-CBP-HA or Flag-HDAC4 expression plasmids. After 36 h of transfection, luciferase activity was measured using the Luciferase Reporter System (Promega), according to the manufacturer's protocol. Relative luciferase activity was calculated by dividing the firefly luciferase activity by the β-gal activity.

### Real-time PCR

Total RNA was isolated from cells using the TRIzol reagent (Invitrogen), according to the manufacturer's protocol. Visualization of the ribosomal bands on a 1% TAE agarose gel was used to assess RNA integrity. cDNA was synthesized from 1 μg of total RNA using the PrimeScript™ RT reagent kit with gDNA Eraser (Takara Biotechnology, Dalian, China). Real-time PCR was carried out using the ABI 7500 Real-time PCR system (Applied Biosystems). The reactions (20 μl) contained cDNA, forward and reverse primers and SYBR Premix Ex Taq™ II (Tli RNaseH Plus) (Takara). The amplification conditions were 95°C for 2 min, followed by 40 cycles of denaturation at 95°C for 15 s, and annealing and extension at 60°C for 1 min. The following primers were used: ALP, Forward: 5′-TGACCTTCTCTCCTC-CATCC-3′, Reverse: 5′-CTTCCTGGGAGTCTCATCCT-3′; Colla 1, Forward: 5′-GCAACAGTCG CTTCACCTACA-3′, Reverse:5′-CAATGTCCAAGGGAGCCACAT-3′; OC, Forward: 5′-TGCTTGTGACGAGCTATCAG-3′, Reverse: 5′-GAGGACAGGGAGGATCAAG T-3′; BSP, Forward: 5′-AAGCAGCACCGTTGAGTATGG-3′; Reverse: 5′-CCTTGTAGTAGCTGTATTCGTCCTC-3′; β-actin, Forward: 5′-AGATGTGGATCAGCAAGCAG-3′, Reverse: 5′-GCGCAAGTTAGGTTTTGTCA-3′. Expression values were normalized to the value for β-actin.

### ChIP assay

ChIP was performed using the EZ-ChIP kit according to the manufacturer's protocol. Briefly, MC3T3 E1 cells were treated with rhBMP-2 (100 ng/ml) for 24 h followed by TSA (20 nM) for 24 h. The cells were then harvested and chemically cross-linked with 1% formaldehyde for 10 min at room temperature. After washing with cold PBS, the cells were lysed, sonicated, and immunoprecipitated with anti-Osx or rabbit-IgG antibodies pre-absorbed with 40 μl of protein A/G beads overnight at 4°C. After several washes, the complexes were eluted and the crosslinking was reversed by incubation at 65°C for 2 h, then extracted (input) and immunoprecipitated DNA was purified. Real-time PCR was performed with the primers corresponded to the promoter regions of *ALP, BSP, Col1a1* and *OC* as follows: ALP promoter primers: forward,5′-CCTGCCTGTTGCAGCCCTACG-3′, reverse,5′-TAAGGAGCGCAGTGGCGGGA-3′; BSP promoter primers: forward, 5′-CTCTTGGCATCAACTCATTTCCTA-3′, reverse, 5′-GAGAAGATGGTAGTTAGAGTTCTG-3′; Col1a1 promoter primers: forward, 5′-AAGGGGTATTCTCTACCCACACTC-3′, reverse, 5′-CAGCCAATCAGAACTGCCTGG-3′; OC promoter primers: forward, 5′-GAGCTGGCAGTCTCCGATTG-3′, reverse, 5′-GCTCTCTGATGTAAGCAGGAGG-3′.

### ALP staining

C2C12 cells seeded in 24-well plates were transfected with RK2-HA-Osx(WT), RK2-HA-Osx^K307R-K312R^ or RK2 empty vector using Lipofectamine 2000. At 24 h after transfection, the cells were treated with BMP2 (100 ng/ml) for 4–5 days. Before staining, the transfected C2C12 cells were fixed in 10% paraformaldehyde for 10 min at room temperature. After washing with PBS, the cells were stained with 300 μg/ml of BCIP/NBT solution for 20 min; alkaline phosphatase-positive cells stained blue/purple.

### Mineralization assay: alizarin red staining (ARS)

C2C12 cells seeded in 24-well plates were transfected with RK2-HA-Osx(WT), RK2-HA-Osx^K307R-K312R^ or RK2 empty vector using Lipofectamine 2000. At 24 h after transfection, the cells were treated with BMP2 (100 ng/ml) for 10–12 days. After the cells were fixed in 5% paraformaldehyde for 10 min, the cells were stained with 2% ARS (pH 7.2) for 15 min and then washed twice with PBS. The orange and red areas were scored as calcium nodules.

### Statistical analysis

Results are expressed as the mean ± S.D. Student's t-test and analysis of variance (ANOVA) were used to assess differences; P<0.05 was considered statistically significant.

## SUPPLEMENTARY MATERIAL FIGURES


