# Supplementary material for: Osterix acetylation at K307 and K312 enhances its transcriptional activity and is required for osteoblast differentiation
Source: Oncotarget. 2016 May 26;7(25):37471–86. doi: 10.18632/oncotarget.9650 (PMC5122325; doi:10.18632/oncotarget.9650)
Supplement: Supplementary file 1 [file oncotarget-07-37471-s001.pdf]

Osterix acetylation at K307 and K312 enhances its transcriptional activity and is required for osteoblast differentiation

Supplementary Material

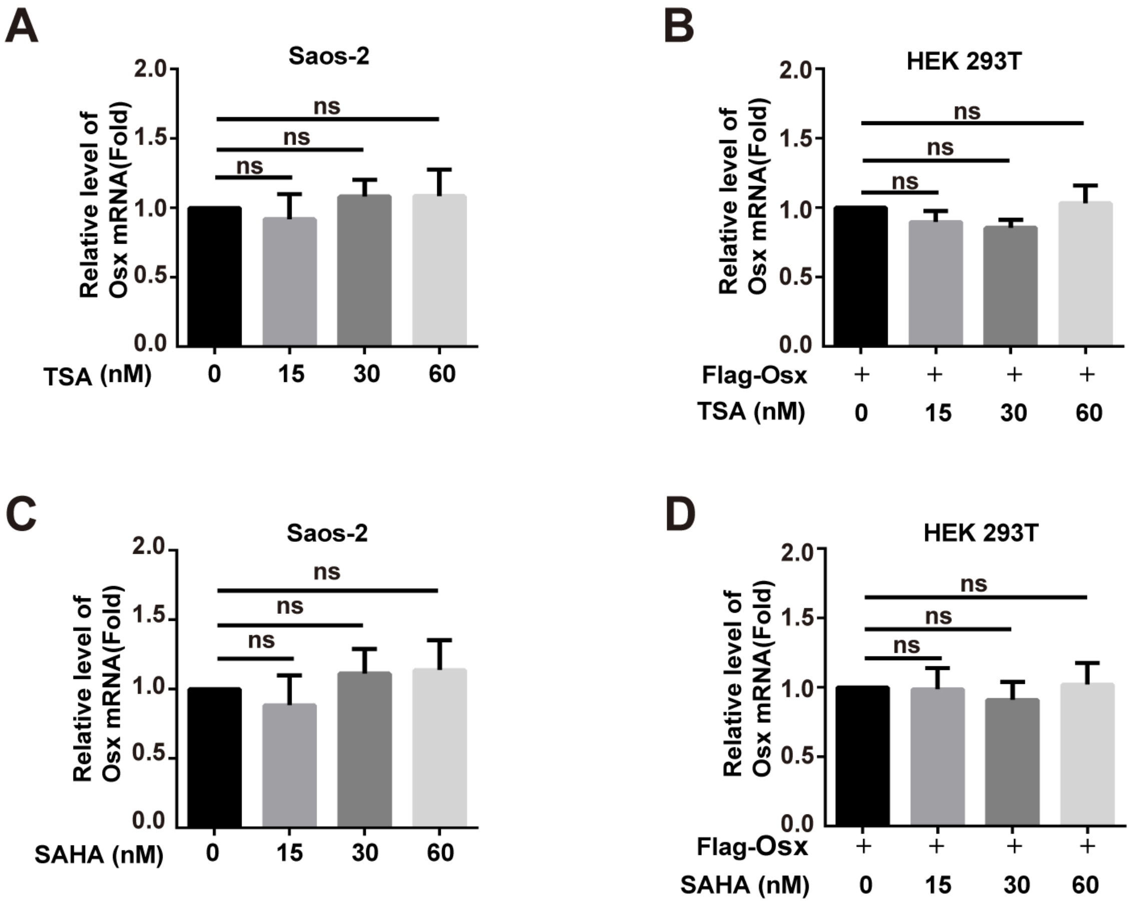

Figure S1. Effects of TSA and SAHA treatment on the mRNA expression of *Osx*. A. Saos-2 cells were treated with TSA (0, 15, 30, 60 nM) for 24 h. B. HEK 293T cells were transfected with Flag-Osx plasmids and then treated with TSA (0, 15, 30, 60 nM) for 24 h. mRNA of *Osx* was detected by real-time PCR. β-Actin was used as an internal control. C. Saos-2 cells were treated with SAHA (0, 15, 30, 60 nM) for 24 h. D. HEK 293T cells were transfected with Flag-Osx plasmids and then treated with SAHA (0, 15, 30, 60 nM) for 24 h. mRNA of Osx was detected by real-time PCR. β-Actin was used as an internal control. The results are from three independent experiments carried out in triplicate. Data are expressed as the mean ± S.D.

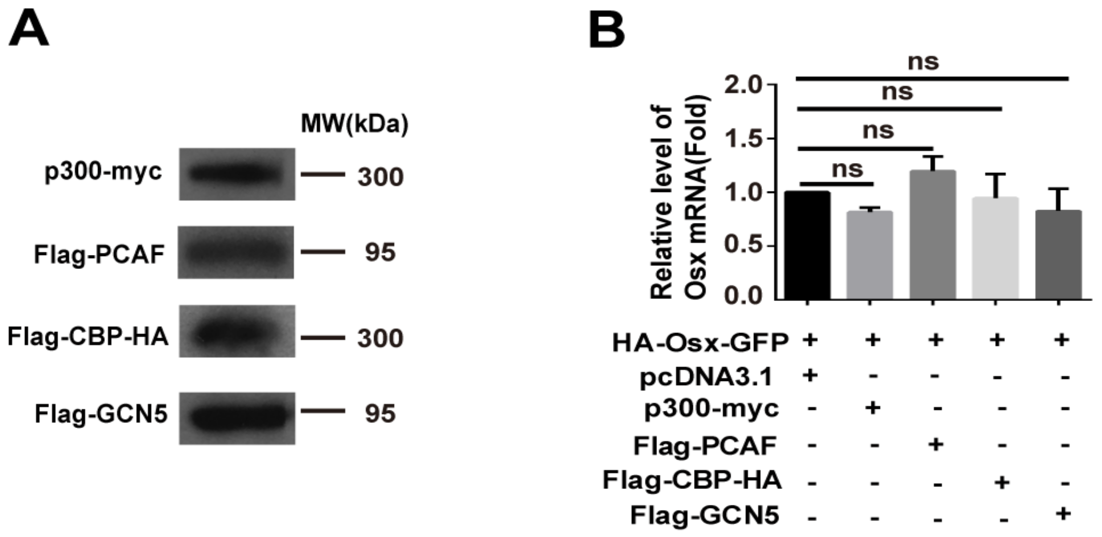

Figure S2. Effects of acetyltransferase on the mRNA expression of *Osx*. HEK 293T cells were transiently co-transfected with HA-Osx-GFP and p300-myc, Flag-PCAF, Flag-CBP-HA, or Flag-GCN5 expression vectors. pcDNA3.1 empty vector co-transfection was used as a control. A. Acetyltransferase protein was detected by western blotting with an anti-Flag or anti-myc antibody. β-Actin served as a loading control. B. mRNA of *Osx* was detected by real-time PCR. β-Actin was use as an internal control. The results are from three independent experiments carried out in triplicate. Data are expressed as the mean ± S.D.

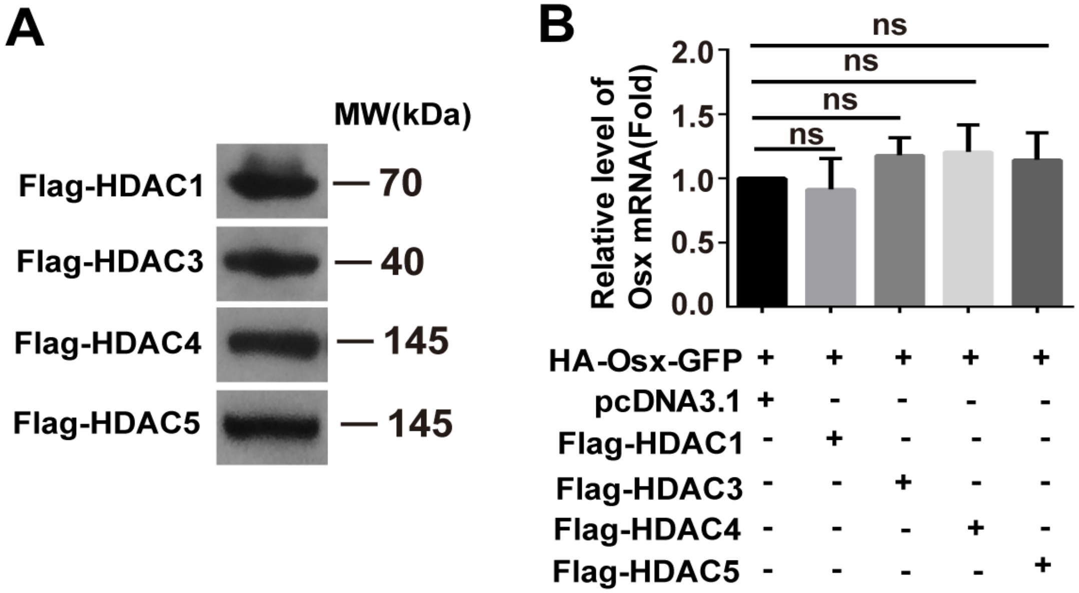

Figure S3. Effects of deacetylase on the mRNA expression of Osx. HEK 293T cells were transiently co-transfected with HA-Osx-GFP and Flag-HDAC1, Flag-HDAC3, Flag-HDAC4, or Flag-HDAC5 expression vectors. pcDNA3.1 empty vector co-transfection was used as a control. A. Deacetylase protein was detected by western blotting with an anti-Flag antibody. β-Actin served as a loading control. B. mRNA of Osx was detected by real-time PCR. β-Actin was used as an internal control. The results are from three independent experiments carried out in triplicate. Data are expressed as the mean ± S.D.

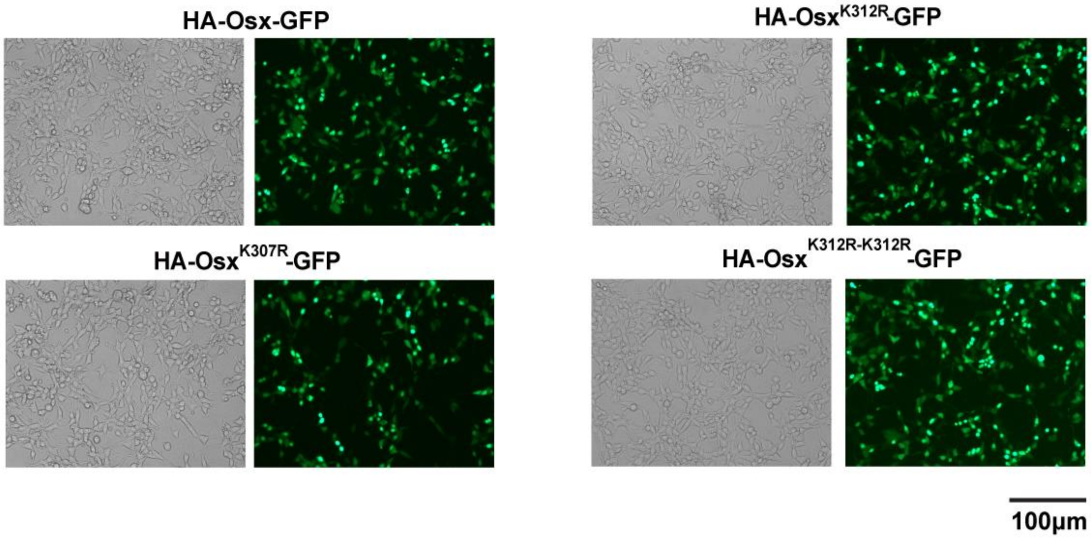

Figure S4. Identification of the transfection efficiency. HEK 293T cells were transfected with HA-Osx-GFP, HA-OsxK307-GFP, HA-OsxK312-GFP and HA-OsxK307-K312-GFP expression plasmids, respectively. 48 h after transfection, the photographs were taken using a fluorescence microscope.

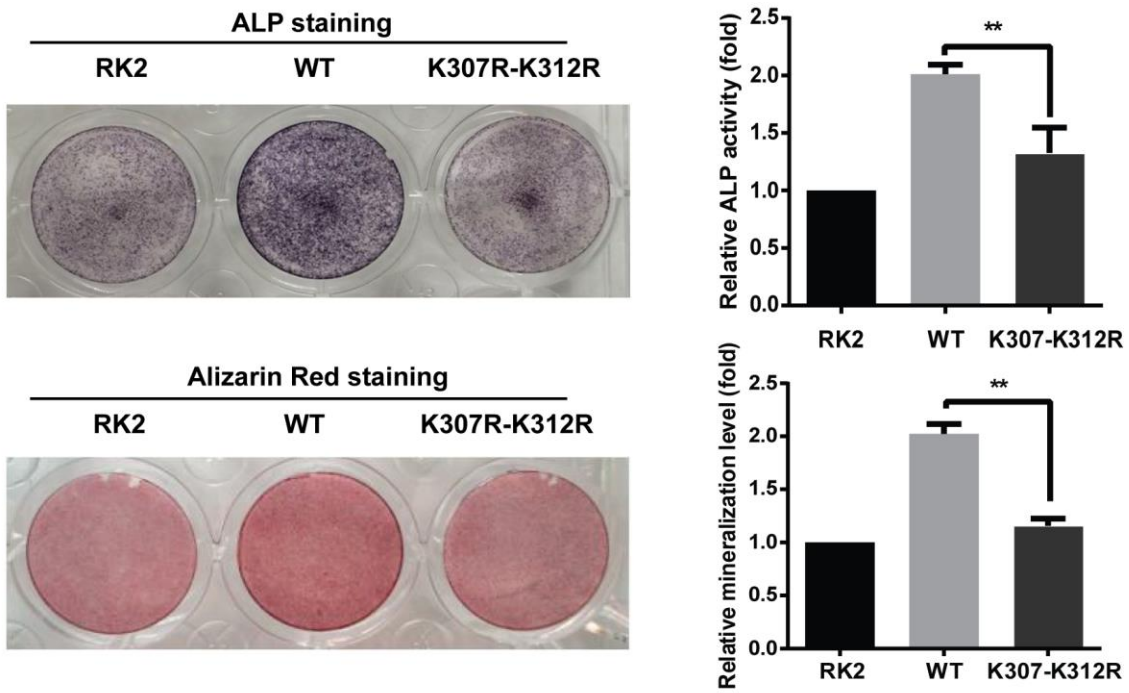

Figure S5. Deacetylation of Osx impairs osteoblast differentiation. MC3T3 E1 cells were transiently transfected with empty RK2 vector, the HA-Osx(WT) expression plasmid or HA-$Osx^{K307R\text{-}K312R}$ mutants. 24 h after transfection, the cells were incubated with BMP2 (100 ng/ml). ALP activity was examined by ALP staining 4–5 days later, or mineralization was assessed using Alizarin Red staining 10–12 days later. Representative images of three independent experiments are shown (left panel). Quantification of ALP activity and mineralization is shown in the right panel. Results are the mean ± S.D. of three independent experiments; ** $p < 0.01$.
